# Supplementary material for: Genome-Wide Study of Conidiation-Related Genes in the Aphid-Obligate Fungal Pathogen Conidiobolus obscurus (Entomophthoromycotina)
Source: J Fungi (Basel). 2022 Apr 12;8(4):389. doi: 10.3390/jof8040389 (PMC9026835; doi:10.3390/jof8040389)

Table S1 The designed primers for qPCR

Internal ID/gene	Primer Sequence	
	Forward	Reverse
mRNAs		
EVM0003971	cGAATATGAGCCTGGAGAT	CGACCTTAGAATCCTTGT
EVM0004814	GATTCTTCGCCGTTCAAG	ACATAAGGGTCTGGTATTCC
EVM0009373	CTCATTGCTGGTGTTGTAT	GTATTACGGTCAAGGATAGAAC
EVM0008905	GACGGACAAGTATGTAATGG	AATGGAAGGAAGTTGAACAG
EF1	CGATACCTCCCTCCGCCCAG	ACAGGCGGAACGACCACAAC

Table S2 Repeative sequence statistic of *Conidiobolus obscurus*

**Type**	**Number**	**Length (bp)**	**Percentage (%)**
Class I	20,908	4,509,867	11.99
Class I/LINE	26	1,523	0.00
Class I/LTR	2	171	0.00
Class I/LTR/Copia	18	1,760	0.00
Class I/LTR/Gypsy	152	304,211	0.81
Class I/PLE|LARD	20,710	4,225,460	11.24
Class II	5,304	968,376	2.57
Class II/MITE	3,362	476,389	1.27
Class II/Maverick	1,527	295,252	0.79
Class II/TIR	193	129,689	0.34
Class II/Unknown	222	93,467	0.25
Potential Host Gene	1,351	259,385	0.69
SSR	25	10,926	0.03
Unknown	9,816	2,088,534	5.55
Total	27,588	6,558,671	17.44

Table S3 Gene number of the protein-encoding genes annotated in different databases

**Database**	**Number**
GO	2,375
KEGG	4,321
KOG	6,465
Pfam	7,710
Swissprot	6,460
TrEMBL	8,195
Nr	8,270
CAZyme	392
TCDB	97
PHI	2,888
all	8,566

Table S4 the sequencing data and alignment statistics

Samples	Clean reads	Clean bases	GC Content*	%≥Q30[Ψ]	Mapped reads
C-1	56,539,926	16,803,169,322	37.10%	94.29%	81,354,935 (71.94%)
C-2	58,566,590	17,455,882,080	37.23%	94.56%	85,057,246 (72.62%)
C-3	60,759,696	18,028,324,124	37.37%	94.66%	75,783,626 (62.36%)
Pre-C-1	21,365,220	6,395,062,008	37.54%	94.11%	35,882,293 (83.97%)
Pre-C-2	20,596,396	6,165,534,548	37.57%	94.24%	34,729,506 (84.31%)
Pre-C-3	21,521,034	6,447,217,940	37.37%	93.94%	36,143,008 (83.79%)
Post-C-1	22,055,956	6,606,661,946	36.81%	94.13%	36,498,667 (82.74%)
Post-C-2	20,455,959	6,128,162,266	36.45%	93.73%	33,753,277 (82.5%)
Post-C-3	22,409,737	6,710,299,490	36.78%	94.48%	37,116,052 (82.81%)

* GC content: GC content percentage of Clean Data, namely the percentage of Clean Data base G and C;

[Ψ] %≥Q30: percentage of Clean Data which quality value is greater than or equal to Q30 (an error rate of 1 in 1000).

Table S5 Differential expression of selected genes putatively related to transcription factors (TF) during conidiation.

Internal ID	FPKM*			Log2(FC) †		PHI	Annotation
	Pre-C	C	Post-C	C vs. pre-C	C vs. post-C		
EVM0007139	0.25	5.57	2.88	3.89	1.21	PHI:287	GATA-4/5/6 TF
EVM0007072	16.24	41.74	23.84	1.39	1.07	/	Histone-like TF
EVM0006849	44.03	101.27	57.09	1.23	1.09	/	Transcription initiation factor TFIIA
Co _242	0.00	18.18	2.89	7.70	2.82	/	Homeobox phx1 TF
EVM0006123	0.92	4.69	0.97	2.33	2.35	PHI:2128	Homeobox phx1 TF
EVM0003245	0.00	2.34	0.13	6.15	3.79	PHI:2039	C2H2 finger domain TF
EVM0001087	1.34	5.91	1.52	2.09	2.12	PHI:2039	C2H2 finger domain TF
EVM0004983	0.06	2.99	0.31	4.95	3.40	PHI:232	STE-like TF
EVM0008462	1.93	18.15	5.54	3.19	1.90	PHI:1405	SteA TF
EVM0002148	0.11	2.13	0.24	3.90	3.18	PHI:3315	Activator of gluconeogenesis TF
EVM0007454	0.33	3.78	0.19	3.44	4.45	PHI:1921	RNA polymerase II-specific TF
EVM0000062	12.39	25.68	11.54	1.09	1.43	PHI:2930	Cutinase TF
EVM0010146	0.13	1.47	0.32	3.10	2.23		Fungal specific TF
EVM0000205	18.17	41.06	15.94	1.22	1.63	PHI:1742	Fungal specific TF
EVM0003677	9.24	111.30	38.21	3.56	1.80	PHI:2565	Heat shock TF
EVM0008905	38.21	269.52	11.23	2.83	4.76	PHI:2406	Heat shock TF
Co _1230	1.00	4.90	0.65	2.16	2.96	/	Pcc1 TF
EVM0008509	28.60	61.05	19.28	1.13	1.93	/	e(y)2 TF
EVM0006858	21.95	71.91	36.99	1.74	1.22	/	Transcription initiation factor TFIID
EVM0002806	13.05	34.55	11.79	1.44	1.82	PHI:1295	Asexual reproduction regulation TF
EVM0000750	98.92	204.70	46.81	1.10	2.36	PHI:3808	CREB family TF

* The transcript level is expressed as fragments per kilobase per million mapped reads (FPKM).

† FC is the fold change (FC) of DEGs based on a FC of ≥ 2 and an FDR of < 0.01.

Table S6 The DEGs associated with the main GO terms

Internal ID	FPKM*			Log2(FC)†		Pfam annotation
	Pre-C	C	Post-C	C vs. pre-C	C vs. post-C	
Biological Process:carbohydrate metabolic process (GO:0005975)						
EVM0003314	0.30	21.02	0.00	5.88	9.51	Glycosyl hydrolases family 16
EVM0005673	0.77	2.40	0.06	1.62	5.33	Glycosyl hydrolases family 31
EVM0009297	25.90	59.04	16.88	1.23	2.07	Glycogen debranching enzyme
EVM0009626	31.33	65.56	20.27	1.11	1.96	Glycogen debranching enzyme
EVM0000365	10.12	47.79	0.87	2.27	5.99	Glycosyl hydrolases family 16
EVM0001373	10.45	48.78	0.90	2.25	5.97	Glycosyl hydrolases family 16
EVM0008569	22.33	49.64	27.33	1.18	1.12	Ribulose-phosphate 3 epimerase family
EVM0007041	76.64	161.17	57.15	1.11	1.76	Ribulose-phosphate 3 epimerase family
EVM0008158	0.00	1.55	0.07	6.09	4.10	Glycosyl hydrolases family 18
EVM0004064	3.64	133.52	22.28	5.14	2.81	Glycosyl hydrolases family 18
EVM0001249	6.24	148.22	24.14	4.48	2.84	Glycosyl hydrolases family 18
EVM0004020	1.58	206.41	17.19	6.98	3.82	Glycosyl hydrolases family 18
EVM0004131	8.18	131.74	23.17	3.99	2.74	Glycosyl hydrolases family 18
Biological Process: transport (GO:0006810)						
EVM0004058	5.71	11.80	5.54	1.08	1.36	ABC transporter
EVM0002255	0.02	3.49	0.42	5.89	2.85	Major intrinsic protein
EVM0006467	0.04	5.42	0.12	4.52	5.47	Sugar (and other) transporter
EVM0007330	2.86	54.35	4.28	4.26	3.92	Sulfate permease family; STAS domain
EVM0006243	1.22	10.98	1.37	3.14	3.23	Sulfate permease family
EVM0009518	12.87	35.39	12.83	1.47	1.68	Sulfate permease family
EVM0009164	7.66	21.62	12.07	1.53	1.10	STAS domain
EVM0002273	4.98	55.02	17.55	3.46	1.92	Sodium/hydrogen

						exchanger family
EVM0004950	23.24	65.43	33.45	1.53	1.24	Mitochondrial carrier
EVM0003315	7.42	16.96	6.98	1.18	1.50	Mitochondrial carrier
EVM0000187	9.02	20.94	8.40	1.26	1.59	ABC transporter
EVM0003819	10.66	20.92	11.24	1.01	1.17	Nucleoside transporter
Biological Process: cellular process (GO:0009987)						
EVM0006180	0.21	7.20	1.11	4.70	2.91	Glucanosyltransferase; X8 domain
EVM0007333	0.14	2.54	0.44	3.80	2.66	Glucanosyltransferase; X8 domain
EVM0004067	86.45	544.69	301.14	2.67	1.12	Cellulase (glycosyl hydrolase family 5)
EVM0010061	3.07	6.70	1.20	1.16	2.68	Protein tyrosine kinase
EVM0008877	8.02	16.60	3.22	1.09	2.61	Protein tyrosine kinase
Co _2537	2.56	5.06	3.09	1.02	1.68	Response regulator receiver domain
EVM0002403	3.76	20.76	10.86	2.48	1.21	Lipase (class 3)
EVM0001310	6.53	15.93	6.64	1.28	1.51	Dolichol phosphate-mannose biosynthesis regulatory protein (DPM2)
EVM0006252	0.00	5.03	0.97	7.84	2.50	Protein tyrosine kinase; Kinase-like
EVM0004435	4.02	24.39	7.19	2.61	2.02	Cyclophilin type peptidyl-prolyl cis-trans isomerase/CLD
EVM0003992	3.91	23.47	6.92	2.59	2.02	Cyclophilin type peptidyl-prolyl cis-trans isomerase/CLD
EVM0003677	9.24	111.30	38.21	3.56	1.80	HSF-type DNA-binding
EVM0002806	13.05	34.55	11.79	1.44	1.82	Cell pattern formation-associated
EVM0003907	0.03	3.64	0.03	3.66	5.64	Peptidase inhibitor I9
EVM0001805	1.07	2.90	0.15	1.44	4.24	Peptidase inhibitor I9
Cellular Component: membrane (GO:0016020)						
EVM0004514	47.08	91.44	40.78	1.01	1.44	Glucanosyltransferase; X8 domain
EVM0002472	2.21	9.34	1.72	2.11	2.71	1,3-beta-glucan synthase
EVM0003159	0.75	7.38	0.34	3.23	4.44	--
EVM0000484	2.27	9.24	1.73	2.06	2.68	1,3-beta-glucan synthase
EVM0004224	2.86	10.57	2.08	1.93	2.61	1,3-beta-glucan synthase
EVM0001764	6.32	21.13	4.13	1.78	2.62	1,3-beta-glucan synthase
EVM0005934	0.01	1.64	0.06	5.13	4.43	Fibronectin type II domain
EVM0004067	86.45	544.69	301.14	2.67	1.12	Cellulase
EVM0010219	126.01	321.21	52.74	1.38	2.87	Fatty acid hydroxylase

						superfamily
EVM0003064	35.03	189.63	16.33	2.47	3.80	ABC transporter
EVM0004058	5.71	11.80	5.54	1.08	1.36	ABC transporter
EVM0000865	0.02	1.09	0.01	5.23	6.10	E1-E2 ATPase
EVM0001785	0.02	1.12	0.01	5.10	6.13	E1-E2 ATPase
EVM0001357	1.69	7.84	0.42	2.22	4.40	E1-E2 ATPase
EVM0005475	23.17	766.40	1.97	5.06	8.74	NADH-ubiquinone reductase complex 1 MLRQ subunit
EVM0006467	0.04	5.42	0.12	4.52	5.47	Major Facilitator Superfamily
EVM0010232	11.82	36.07	3.93	1.65	3.45	Sugar (and other) transporter
EVM0004950	23.24	65.43	33.45	1.53	1.24	Mitochondrial carrier protein
EVM0004653	50.58	124.17	52.37	1.28	1.47	Mitochondrial carrier protein
EVM0003315	7.42	16.96	6.98	1.18	1.50	Mitochondrial carrier protein
EVM0004218	2.40	11.34	3.28	2.17	1.98	Heavy-metal-associated domain
EVM0005466	28.92	90.54	17.56	1.69	2.64	EamA-like transporter family
EVM0006025	11.59	27.57	5.91	1.29	2.49	EamA-like transporter family
EVM0000187	9.02	20.94	8.40	1.26	1.59	ABC transporter
EVM0005299	69.05	137.92	39.23	1.01	2.08	Cytochrome c oxidase subunit VIc
EVM0002609	3.71	22.25	7.96	2.61	1.75	Glycosyl hydrolase family 47
EVM0003938	19.16	94.08	24.69	2.30	2.17	NADH-ubiquinone oxidoreductase MWFE subunit
Cellular Component: cytosol (GO:0005829)						
EVM0001685	0.23	10.18	0.19	5.06	5.52	Ubiquitin-conjugating enzyme
Co_1850	7.65	24.25	8.56	1.54	1.61	LSM domain
EVM0005642	6.97	27.14	10.29	1.98	1.65	LSM domain
EVM0002852	11.65	33.69	11.81	1.56	1.77	Ubiquitin family
EVM0000395	72.33	192.18	69.85	1.44	1.73	Ubiquitin family
EVM0010225	12.44	29.32	6.67	1.25	2.33	U1 zinc finger
EVM0002141	7.76	15.95	4.99	1.07	1.93	Cytidine and deoxycytidylate deaminase zinc-binding region

EVM0009693	2.90	18.26	3.28	2.54	2.62	Uncharacterised protein family
EVM0005733	887.99	2628.51	612.76	1.60	2.36	Ribosomal L29 protein
EVM0006554	50.75	259.70	60.76	2.38	2.35	Thioredoxin; AhpC/TSA family
EVM0002020	99.80	266.43	113.85	1.44	1.49	Thioredoxin; AhpC/TSA family
EVM0002444	252.14	506.80	158.18	1.03	1.94	Thioredoxin; AhpC/TSA family
EVM0003561	89.95	228.12	117.78	1.36	1.21	EF-hand domain
EVM0004218	2.40	11.34	3.28	2.17	1.98	Heavy-metal-associated domain
EVM0006741	4.89	11.72	5.87	1.25	1.23	Urm1 (Ubiquitin related modifier)
EVM0000925	15.56	44.95	18.82	1.57	1.53	Acyl transferase
EVM0001674	12.36	34.64	13.94	1.53	1.59	Acyl transferase
EVM0001137	10.84	25.60	9.36	1.26	1.72	Acyl transferase
Cellular Component: nucleus (GO:0005634)						
EVM0001685	0.23	10.18	0.19	5.06	5.52	Ubiquitin-conjugating enzyme
EVM0002852	11.65	33.69	11.81	1.56	1.77	Ubiquitin family
EVM0000395	72.33	192.18	69.85	1.44	1.73	Ubiquitin family
EVM0009693	2.90	18.26	3.28	2.54	2.62	Uncharacterised protein family
EVM0006554	50.75	259.70	60.76	2.38	2.35	Thioredoxin
EVM0002020	99.80	266.43	113.85	1.44	1.49	Thioredoxin
EVM0002444	252.14	506.80	158.18	1.03	1.94	Thioredoxin
EVM0003561	89.95	228.12	117.78	1.36	1.21	EF-hand domain
EVM0004218	2.40	11.34	3.28	2.17	1.98	Heavy-metal-associated domain
EVM0010037	177.80	1749.36	115.71	3.31	4.18	Acyl CoA binding protein
EVM0008569	22.33	49.64	27.33	1.18	1.12	Ribulose-phosphate 3 epimerase
EVM0007041	76.64	161.17	57.15	1.11	1.76	Ribulose-phosphate 3 epimerase
EVM0004983	0.06	2.99	0.31	4.95	3.40	STE like transcription factor
EVM0002148	0.11	2.13	0.24	3.90	3.18	Fungal Zn(2)-Cys(6) binuclear cluster domain
Molecular Function: hydrolase activity (GO:0016787)						
EVM0009290	5.10	251.35	26.34	5.64	3.50	Cellulase
EVM0007726	28.46	63.04	8.57	1.19	3.13	Glycosyl hydrolase family
EVM0004067	86.45	544.69	301.14	2.67	1.12	Cellulase)
EVM0003971	42.26	105.31	50.33	1.36	1.34	Beta-glucan synthesis-

						associated protein (SKN1)
Co_1182	0.00	0.47	0.11	6.18	2.22	Platelet-activating factor acetylhydrolase
EVM0002220	1.39	6.54	1.47	2.26	2.41	Amidohydrolase family
EVM0000082	0.02	0.65	0.00	4.10	6.64	Subtilase family
EVM0001805	1.07	2.90	0.15	1.44	4.24	Subtilase family
EVM0008226	9.05	53.10	1.22	2.57	5.63	Trypsin
EVM0005001	21.39	88.68	1.10	2.08	6.55	Trypsin
EVM0009772	2.42	5.80	0.08	1.27	5.66	Trypsin
EVM0001721	48.83	147.41	19.30	1.63	3.20	Trypsin
EVM0000789	0.02	2.06	0.01	5.13	6.18	Glycosyl hydrolases family 18
EVM0000088	0.47	10.13	0.64	4.38	4.18	Glycosyl hydrolases family 18
EVM0007380	0.00	1.05	0.01	5.72	5.23	Glycosyl hydrolases family 18
EVM0000612	0.46	8.30	0.60	4.13	4.00	Glycosyl hydrolases family 18
EVM0008574	0.11	0.87	0.27	2.72	1.88	Glycosyl hydrolases family 18
EVM0006427	0.11	0.84	0.32	2.68	1.63	Glycosyl hydrolases family 18
EVM0003563	0.11	0.84	0.25	2.64	1.89	Glycosyl hydrolases family 18
Molecular Function: catalytic activity (GO:0003824)						
EVM0006541	0.06	220.04	63.88	9.69	2.04	Polysaccharide deacetylase
EVM0008805	0.00	10.70	0.00	8.66	9.19	Glycosyl hydrolases family 16
EVM0008271	0.00	9.93	0.00	8.44	8.98	Glycosyl hydrolases family 16
EVM0004224	2.86	10.57	2.08	1.93	2.61	1,3-beta-glucan synthase
EVM0000444	0.88	28.13	1.34	5.02	4.65	Chitin synthase
EVM0006157	0.01	6.72	0.74	5.96	3.32	Pyridoxal-dependent decarboxylase
EVM0009297	25.90	59.04	16.88	1.23	2.07	Glycogen debranching enzyme
EVM0009626	31.33	65.56	20.27	1.11	1.96	Glycogen debranching enzyme
EVM0003949	0.92	6.97	2.76	2.80	1.56	HD domain
EVM0003025	10.15	24.96	14.34	1.31	1.05	AMP-binding enzyme
Co_102	0.03	4.79	0.04	6.32	6.31	Eukaryotic aspartyl protease
EVM0007192	43.88	125.58	33.11	1.56	2.17	Aminotransferase class I and II

EVM0007255	6.16	17.73	7.03	1.55	1.60	Glycerophosphoryl diester phosphodiesterase family
EVM0004087	7.96	49.50	7.38	2.57	2.99	Aminotransferase class I and II
Molecular Function: serine-type peptidase activity (GO:0008236)						
EVM0007218	0.00	6.15	0.01	7.49	7.29	Subtilase family
EVM0000403	0.67	25.11	1.45	3.77	3.65	Subtilase family
EVM0005632	0.41	10.97	1.22	3.70	2.98	Subtilase family
EVM0004644	147.47	970.44	12.14	2.74	6.57	Subtilase family
EVM0008109	0.06	14.77	0.12	6.93	4.37	Trypsin
EVM0006838	4.46	290.36	7.64	5.96	5.43	Trypsin
EVM0008156	0.47	36.30	3.51	5.95	3.56	Trypsin
EVM0007680	0.16	8.65	0.04	5.14	6.88	Trypsin
EVM0006920	10.03	255.93	10.92	4.64	4.76	Trypsin
Co_2766	2.87	42.32	0.45	3.87	6.62	Trypsin
EVM0009083	5.02	36.42	0.99	2.87	5.41	Trypsin
EVM0003166	6.32	27.37	6.54	2.15	2.33	Trypsin

* The transcript level is expressed in fragments per kilobase per million fragments (FPKM) values. C: mycelia at sporulating stage; Pre-C: mycelia before sporulating stage; Post-C: mycelia after sporulating stage.

† FC is the fold change of differentially expressed genes between the three libraries of C, Pre-C and PostC. There are genes expressing with significant difference (a fold change ≥ 2 and a false discovery rate < 0.001).

Table S7 The DEGs associated with the main KEGG pathway

Internal ID	FPKM*			Log2(FC)†		Pfam annotation
	Pre-C	C	Post-C	C vs. pre-C	C vs. post-C	
Oxidative phosphorylation (ko00190)						
EVM0009508	19.49	101.99	24.80	2.40	2.30	ATP synthase subunit H
EVM0004390	11.36	41.00	12.96	1.86	1.91	Vacuolar (H+)-ATPase G subunit
EVM0000869	11.09	39.75	12.80	1.85	1.88	Vacuolar (H+)-ATPase G subunit
EVM0000557	85.43	262.37	75.67	1.65	2.05	Vacuolar (H+)-ATPase G subunit
EVM0005475	23.17	766.40	1.97	5.06	8.74	NADH-ubiquinone reductase complex 1
EVM0007673	82.11	222.07	41.72	1.45	2.65	NADH-ubiquinone oxidoreductase B18
EVM0004399	40.00	104.82	37.18	1.39	1.73	NADH:ubiquinone oxidoreductase,
EVM0001245	45.65	241.07	37.97	2.42	2.92	Cytochrome oxidase c subunit VIb
EVM0008584	20.72	62.39	11.85	1.61	2.64	Cytochrome c oxidase subunit VIIc
EVM0004161	50.11	185.06	101.63	1.87	1.11	Cytochrome oxidase assembly protein
EVM0006582	254.08	559.04	183.56	1.17	1.88	Cytochrome c oxidase subunit VIa
EVM0005299	69.05	137.92	39.23	1.01	2.08	Cytochrome c oxidase subunit VIc
EVM0008956	8.39	39.48	8.45	2.20	2.44	Ubiquinol-cytochrome C reductase hinge protein
EVM0007884	9.44	36.04	10.39	1.93	2.04	Ubiquinol-cytochrome C reductase hinge protein
EVM0008409	9.62	34.39	10.52	1.81	1.93	Ubiquinol-cytochrome C reductase hinge protein
EVM0002948	16.20	66.97	14.73	2.02	2.41	Ubiquinol-cytochrome C reductase
EVM0003754	11.42	47.32	10.64	1.99	2.35	Ubiquinol-cytochrome C reductase
EVM0009982	11.29	26.73	13.52	1.25	1.22	Ubiquinol-cytochrome-

						c reductase complex subunit (QCR10)
EVM0005826	37.87	139.64	36.72	1.89	2.19	ATP synthase complex subunit h
EVM0008514	58.16	164.13	46.60	1.52	2.07	ATP synthase complex subunit h
EVM0000544	63.03	231.60	29.49	1.88	3.20	Mitochondrial ATP synthase epsilon chain
EVM0001330	39.78	120.09	32.18	1.61	2.15	Mitochondrial ribosomal protein L51 / S25 / CI-B8 domain
EVM0005601	42.32	109.17	29.39	1.37	2.13	ATP synthase E chain
**Amino sugar and nucleotide sugar metabolism (ko00520)**						
EVM0000444	0.88	28.13	1.34	5.02	4.65	Chitin synthase
EVM0004669	3.74	36.43	11.22	3.31	1.97	Chitin synthase
EVM0001244	48.91	588.98	30.89	3.63	4.52	Glycosyl hydrolase family 20
EVM0005566	74.65	342.19	19.81	2.23	4.37	Glycosyl hydrolase family 20
EVM0007277	20.04	42.44	11.52	1.12	2.14	Acetyltransferase (GNAT) family
EVM0008158	0.00	1.55	0.07	6.09	4.10	Glycosyl hydrolases family 18
EVM0000789	0.02	2.06	0.01	5.13	6.18	Glycosyl hydrolases family 18
EVM0007628	1.09	27.49	0.76	4.65	5.38	Glycosyl hydrolases family 18
EVM0000088	0.47	10.13	0.64	4.38	4.18	Glycosyl hydrolases family 18
EVM0007380	0.00	1.05	0.01	5.72	5.23	Glycosyl hydrolases family 18
EVM0004064	3.64	133.52	22.28	5.14	2.81	Glycosyl hydrolases family 18
EVM0001249	6.24	148.22	24.14	4.48	2.84	Glycosyl hydrolases family 18
EVM0004020	1.58	206.41	17.19	6.98	3.82	Glycosyl hydrolases family 18
EVM0004131	8.18	131.74	23.17	3.99	2.74	Glycosyl hydrolases family 18
EVM0002377	3.67	21.22	3.63	2.56	2.81	Glycosyl hydrolases family 18
EVM0000612	0.46	8.30	0.60	4.13	4.00	Glycosyl hydrolases family 18
EVM0008574	0.11	0.87	0.27	2.72	1.88	Glycosyl hydrolases

						family 18
EVM0006427	0.11	0.84	0.32	2.68	1.63	Glycosyl hydrolases family 18
EVM0003563	0.11	0.84	0.25	2.64	1.89	Glycosyl hydrolases family 18
EVM0005693	69.41	186.04	94.25	1.45	1.25	GDP-mannose 4,6 dehydratase
**MAPK signaling pathway - yeast (ko04011)**						
EVM0008074	0.21	1.79	0.08	2.84	4.10	1,3-beta-glucan synthase component
EVM0002472	2.21	9.34	1.72	2.11	2.71	1,3-beta-glucan synthase component
EVM0003159	0.75	7.38	0.34	3.23	4.44	1,3-beta-glucan synthase component
EVM0007566	4.93	23.19	3.05	2.26	3.18	1,3-beta-glucan synthase component
EVM0000484	2.27	9.24	1.73	2.06	2.68	1,3-beta-glucan synthase component
EVM0004224	2.86	10.57	2.08	1.93	2.61	1,3-beta-glucan synthase component
EVM0001764	6.32	21.13	4.13	1.78	2.62	1,3-beta-glucan synthase component
EVM0005361	18.15	309.79	148.14	4.10	1.32	Catalase-related immune-responsive
EVM0009987	4.35	71.98	34.28	4.02	1.33	Catalase-related immune-responsive
EVM0007860	6.31	102.14	50.99	3.99	1.26	Catalase-related immune-responsive
EVM0001653	22.96	61.12	33.62	1.45	1.13	Variant SH3 domain
EVM0004983	0.06	2.99	0.31	4.95	3.40	STE like transcription factor
EVM0001126	5.85	71.98	23.78	3.62	1.86	GGL domain
EVM0008649	126.30	916.02	323.30	2.89	1.77	GGL domain
EVM0000750	98.92	204.70	46.81	1.10	2.36	bZIP transcription factor
**Ribosome (ko03010)**						
EVM0000242	815.77	2264.82	654.09	1.51	2.06	S25 ribosomal protein
EVM0002937	950.39	3871.05	526.06	2.05	3.14	Ribosomal protein S28e
EVM0005588	1245.76	3090.38	851.98	1.34	2.13	Ribosomal protein S27
EVM0009766	3.72	12.37	5.28	1.69	1.46	Ribosomal protein L36
EVM0009938	4.64	13.08	6.88	1.46	1.16	Ribosomal protein L34
EVM0003011	1717.35	7151.17	1263.06	2.09	2.77	Ribosomal protein L44
Co_61	110.67	363.52	56.75	1.74	2.93	Ribosomal L38e protein family

EVM0005733	887.99	2628.51	612.76	1.60	2.36	Ribosomal L29 protein
Co_2197	79.87	185.90	47.97	1.25	2.22	Ribosomal L39 protein
Co_3023	210.28	485.31	155.24	1.24	1.91	Ribosomal L38e protein family
EVM0008541	286.98	658.84	211.23	1.23	1.91	Ribosomal L38e protein family
EVM0001436	2710.86	6189.85	1919.84	1.23	1.96	Ribosomal protein L32
EVM0007727	124.99	359.92	71.21	1.56	2.60	Ribosomal protein S27a
EVM0002763	750.10	1656.68	494.70	1.17	2.01	Ribosomal protein S27a

* The transcript level is expressed in fragments per kilobase per million fragments (FPKM) values. C: mycelia at sporulating stage; Pre-C: mycelia before sporulating stage; Post-C: mycelia after sporulating stage.

† FC is the fold change of differentially expressed genes between the three libraries of C, Pre-C and Post-C. There are genes expressing with significant difference (a fold change ≥ 2 and a false discovery rate < 0.001).

Table S8 Differential expression of selected genes related to cell wall/membrane/envelope biogenesis during conidiation.

Internal ID	FPKM*			Log2(FC) †		PHI
	Pre-C	C	Post-C	C vs. pre-C	C vs. post-C	
**1,3-beta-glucan synthase**						
EVM0008074	0.21	1.79	0.08	2.84	4.10	PHI:2533
EVM0002472	2.21	9.34	1.72	2.11	2.71	PHI:2533
EVM0003159	0.75	7.38	0.34	3.23	4.44	PHI:2533
EVM0007566	4.93	23.19	3.05	2.26	3.18	PHI:2533
EVM0000484	2.27	9.24	1.73	2.06	2.68	PHI:2533
EVM0004224	2.86	10.57	2.08	1.93	2.61	PHI:2533
EVM0001764	6.32	21.13	4.13	1.78	2.62	PHI:2533
EVM0003971	42.26	105.31	50.33	1.36	1.34	/
**Glucanosyltransferase**						
EVM0006180	0.21	7.20	1.11	4.70	2.91	PHI:33
EVM0007333	0.14	2.54	0.44	3.80	2.66	PHI:33
EVM0004514	47.08	91.44	40.78	1.01	1.44	PHI:33
**Glycosyl hydrolase**						
EVM0007001	4.14	54.19	23.03	3.73	1.51	/
EVM0007042	32.83	87.13	11.42	1.45	3.19	PHI:748
EVM0007726	28.46	63.04	8.57	1.19	3.13	PHI:748
EVM0005673	0.77	2.40	0.06	1.62	5.33	PHI:1071
EVM0003793	0.10	1.22	0.03	3.10	4.73	/
**Chitin synthase/chitinase**						
EVM0000444	0.88	28.13	1.34	5.02	4.65	PHI:3048
EVM0004669	3.74	36.43	11.22	3.31	1.97	PHI:3050
EVM0008158	0.00	1.55	0.07	6.09	4.10	PHI:2388
EVM0000789	0.02	2.06	0.01	5.13	6.18	PHI:2388
EVM0007628	1.09	27.49	0.76	4.65	5.38	PHI:2388
EVM0000088	0.47	10.13	0.64	4.38	4.18	PHI:2388
**Cellulase**						
EVM0009290	5.10	251.35	26.34	5.64	3.50	PHI:323
EVM0004067	86.45	544.69	301.14	2.67	1.12	PHI:323
**Ergosterol**						
EVM0010219	126.01	321.21	52.74	1.38	2.87	PHI:3035
**Mannan biosynthesis/mannoprotein assembly**						
EVM0006408	0.47	277.15	40.64	8.94	2.98	/
EVM0003767	0.95	5.10	2.14	2.35	1.48	PHI:2482

* The transcript level is expressed as fragments per kilobase per million mapped reads (FPKM).

† FC is the fold change (FC) of DEGs based on a FC of ≥ 2 and an FDR of < 0.01.

Table S9 The DEGs associated with PHI database*

DEG ID	PHI
EVM0001654	PHI:432|FGL1|AAQ23181|5518|Fusarium_graminearum|Reduced_virulence
EVM0003421	AKT7|PHI:4194|V5XZS6|5599|Alternaria_alternata|increased_virulence_(Hypervirulence)
EVM0006252	PHI:1221|FGSG_03146|I1RJM9|5518|Gibberella_zeae_(related:_Fusarium_graminearum)|Lethal
EVM0004341	PHI:2117|SPM1|P58371|148305|Magnaporthe_oryzae_(related:_Magnaporthe_grisea)|Reduced_virulence
EVM0004394	AKT7|PHI:4194|V5XZS6|5599|Alternaria_alternata|increased_virulence_(Hypervirulence)
EVM0007784	PHI:432|FGL1|AAQ23181|5518|Fusarium_graminearum|Reduced_virulence
EVM0007218	PHI:2117|SPM1|P58371|148305|Magnaporthe_oryzae_(related:_Magnaporthe_grisea)|Reduced_virulence
EVM0009119	PHI:652|GIP1|AAL11720|67593|Phytophthora_sojae|Effector_(plant_avirulence_determinant)
EVM0004379	AKT7|PHI:4194|V5XZS6|5599|Alternaria_alternata|increased_virulence_(Hypervirulence)
EVM0004020	PHI:144|CHT42|AAC05829|29875|Trichoderma_virens|Reduced_virulence
EVM0008109	PHI:653|GIP2|AAL11721|67593|Phytophthora_sojae|Effector_(plant_avirulence_determinant)
EVM0008303	PHI:2321|SidI|Q4WR83|746128|Aspergillus_fumigatus|Reduced_virulence
EVM0009549	AKT7|PHI:4194|V5XZS6|5599|Alternaria_alternata|increased_virulence_(Hypervirulence)
EVM0002185	AKT7|PHI:4194|V5XZS6|5599|Alternaria_alternata|increased_virulence_(Hypervirulence)
EVM0000830	PHI:1180|(Sc_Sat4)|I1RS32|5518|Gibberella_zeae_(related:_Fusarium_graminearum)|Reduced_virulence
EVM0003125	PHI:2042|ABC3|Q3Y5V5|148305|Magnaporthe_oryzae_(related:_Magnaporthe_grisea)|Loss_of_pathogenicity
EVM0002496	PHI:652|GIP1|AAL11720|67593|Phytophthora_sojae|Effector_(plant_avirulence_determinant)
EVM0009502	PHI:438|BcBOT1_(related:_CND5)|AAQ16576|40559|Botrytis_cinerea|Reduced_virulence
EVM0004492	PHI:397|LAC2|AAV64894|5207|Cryptococcus_neoformans|Unaffected_pathogenicity
EVM0008865	PHI:429|ClPLS1|CAD43407|290576|Colletotrichum_lindemuthianum|Loss_of_pathogenicity
EVM0001751	PHI:2117|SPM1|P58371|148305|Magnaporthe_oryzae_(related:_Magnaporthe_grisea)|Reduced_virulence

EVM0003245	PHI:2039|Con7p|Q069J4|148305|Magnaporthe_oryzae_(related:_Magnaporthe_grisea)|Loss_of_pathogenicity
EVM0008158	PHI:2388|Chi2|0|5530|Metarhizium_anisopliae|Mixed_outcome
EVM0006838	PHI:652|GIP1|AAL11720|67593|Phytophthora_sojae|Effector_(plant_avirulence_determinant)
EVM0006157	PHI:177|ODC|CAB56523|13684|Stagonospora_nodorum|Reduced_virulence
EVM0008156	PHI:652|GIP1|AAL11720|67593|Phytophthora_sojae|Effector_(plant_avirulence_determinant)
EVM0000614	PHI:697|ugt51E1|AAM81358|5022|Leptosphaeria_maculans|Unaffected_pathogenicity
EVM0001824	PHI:1161|MgMfs1|A4ZGP3|54734|Mycosphaerella_graminicola_(related:_Zymoseptoria_tritici)|Chemistry_target
EVM0007380	PHI:144|CHT42|AAC05829|29875|Trichoderma_virens|Reduced_virulence
EVM0009290	PHI:323|VFGLU1|AAO63562|93591|Verticillium_fungicola|Reduced_virulence
EVM0004016	PHI:511|CaNAG4|EAK93098|5476|Candida_albicans|Reduced_virulence
EVM0008454	PHI:1541|GzFlbD|I1RE42|5518|Gibberella_zeae_(related:_Fusarium_graminearum)|Unaffected_pathogenicity
EVM0000952	PHI:777|MGG_00883|EDK02450|318829|Magnaporthe_oryzae|Loss_of_pathogenicity
EVM0002960	AKT7|PHI:4194|V5XZS6|5599|Alternaria_alternata|increased_virulence_(Hypervirulence)
EVM0007590	PHI:2021|Pac2|MGG_06564|5270|Ustilago_maydis|Reduced_virulence
EVM0005882	Ktr1|PHI:3095|J5JDI1|176275|Beauveria_bassiana|unaffected_pathogenicity_
EVM0001943	PHI:577|NoxB|BAE72682|35717|Epichloe_festucae|Wild-type_mutualism
EVM0000865	PHI:440|PMR1|CAB87245|5476|Candida_albicans|Reduced_virulence
EVM0007554	PHI:2117|SPM1|P58371|148305|Magnaporthe_oryzae_(related:_Magnaporthe_grisea)|Reduced_virulence
EVM0004064	PHI:144|CHT42|AAC05829|29875|Trichoderma_virens|Reduced_virulence
EVM0007680	PHI:653|GIP2|AAL11721|67593|Phytophthora_sojae|Effector_(plant_avirulence_determinant)
EVM0000789	PHI:2388|Chi2|0|5530|Metarhizium_anisopliae|Mixed_outcome
EVM0001785	PHI:440|PMR1|CAB87245|5476|Candida_albicans|Reduced_virulence
EVM0000444	CSMA|PHI:3048|Q4X0H6|746128|Aspergillus_fumigatus|mixed_outcome_
EVM0004983	PHI:232|STE12a|AAG49739|5207|Cryptococcus_neoformans|Reduced_virulence

EVM0005502	PHI:1511|GzHOME009|I1RXF3|5518|Gibberella_zeae_(related:_Fusarium_graminearum)|Reduced_virulence
EVM0007786	PHI:1221|FGSG_03146|I1RJM9|5518|Gibberella_zeae_(related:_Fusarium_graminearum)|Lethal
EVM0003715	PHI:2357|CYP52X1|E2EAF6|475271|Beauveria_bassiana|Reduced_virulence
EVM0009373	PHI:652|GIP1|AAL11720|67593|Phytophthora_sojae|Effector_(plant_avirulence_determinant)
EVM0006180	PHI:33|PHR1|AAA68196|5476|Candida_albicans|Reduced_virulence
EVM0007628	PHI:2388|Chi2|0|5530|Metarhizium_anisopliae|Mixed_outcome
EVM0006920	PHI:653|GIP2|AAL11721|67593|Phytophthora_sojae|Effector_(plant_avirulence_determinant)
EVM0006467	PHI:2968|Hxs1|J9VQA5|5207|Cryptococcus_neoformans|Reduced_virulence
EVM0001249	PHI:144|CHT42|AAC05829|29875|Trichoderma_virens|Reduced_virulence
EVM0001327	AKT7|PHI:4194|V5XZS6|5599|Alternaria_alternata|increased_virulence_(Hypervirulence)
EVM0004130	PHI:1434|GzC2H103|I1S8Y5|5518|Gibberella_zeae_(related:_Fusarium_graminearum)|Unaffected_pathogenicity
EVM0000088	PHI:2388|Chi2|0|5530|Metarhizium_anisopliae|Mixed_outcome
EVM0008112	PHI:2839|RED1|Q8NJQ2|5016|Cochliobolus_heterostrophus|Reduced_virulence
EVM0000612	PHI:144|CHT42|AAC05829|29875|Trichoderma_virens|Reduced_virulence
EVM0003018	PHI:2839|RED1|Q8NJQ2|5016|Cochliobolus_heterostrophus|Reduced_virulence
EVM0005361	PHI:106|CAT1|AAC39448|5476|Candida_albicans|Reduced_virulence
EVM0007723	PHI:104|CaMNT1|CAA67930|5476|Candida_albicans|Reduced_virulence
EVM0009987	PHI:106|CAT1|AAC39448|5476|Candida_albicans|Reduced_virulence
EVM0004131	PHI:144|CHT42|AAC05829|29875|Trichoderma_virens|Reduced_virulence
EVM0007860	PHI:106|CAT1|AAC39448|5476|Candida_albicans|Reduced_virulence
EVM0001410	PHI:1310|GzbHLH011|I1RQC1|5518|Gibberella_zeae_(related:_Fusarium_graminearum)|Unaffected_pathogenicity
EVM0010093	PHI:432|FGL1|AAQ23181|5518|Fusarium_graminearum|Reduced_virulence
EVM0002148	conx1|PHI:3315|G4MT41|318829|Magnaporthe_oryzae|reduced_virulence_
EVM0007672	PHI:2839|RED1|Q8NJQ2|5016|Cochliobolus_heterostrophus|Reduced_virulence

EVM0007139	PHI:287|CLNR1|AAN65464|290576|Colletotrichum_lindemuthianum|Loss_of_pathogenicity
EVM0007333	PHI:33|PHR1|AAA68196|5476|Candida_albicans|Reduced_virulence
EVM0000403	PHI:2117|SPM1|P58371|148305|Magnaporthe_oryzae_(related:_Magnaporthe_grisea)|Reduced_virulence
EVM0007619	PHI:2117|SPM1|P58371|148305|Magnaporthe_oryzae_(related:_Magnaporthe_grisea)|Reduced_virulence
EVM0005600	PHI:2920|FET3-1|E3QRA4|31870|Colletotrichum_graminicola|Reduced_virulence
EVM0004577	PHI:383|SOD5|EAL00626|5476|Candida_albicans|Loss_of_pathogenicity
EVM0005632	PHI:2117|SPM1|P58371|148305|Magnaporthe_oryzae_(related:_Magnaporthe_grisea)|Reduced_virulence
EVM0006099	PHI:213|CPA1|AAF69795|5207|Cryptococcus_neoformans|Reduced_virulence
EVM0003907	PHI:2117|SPM1|P58371|148305|Magnaporthe_oryzae_(related:_Magnaporthe_grisea)|Reduced_virulence
EVM0001001	PHI:652|GIP1|AAL11720|67593|Phytophthora_sojae|Effector_(plant_avirulence_determinant)
EVM0003677	PHI:2565|Q5APJ0|Q5APJ0|5476|Candida_albicans|Reduced_virulence
EVM0003126	PHI:1161|MgMfs1|A4ZGP3|54734|Mycosphaerella_graminicola_(related:_Zymoseptoria_tritici)|Chemistry_target
EVM0009891	PHI:674|Can2|AAZ30051|178876|Cryptococcus_neoformans|Increased_virulence_(Hypervirulence)
EVM0001772	PHI:2839|RED1|Q8NJQ2|5016|Cochliobolus_heterostrophus|Reduced_virulence
EVM0009087	PHI:2128|MoHox1|MGG_04853|148305|Magnaporthe_oryzae_(related:_Magnaporthe_grisea)|reduced_virulence
EVM0006347	PHI:2844|BRM2|O93802|5599|Alternaria_alternata|Unaffected_pathogenicity
EVM0005592	AKT7|PHI:4194|V5XZS6|5599|Alternaria_alternata|increased_virulence_(Hypervirulence)
EVM0002733	PHI:2844|BRM2|O93802|5599|Alternaria_alternata|Unaffected_pathogenicity
EVM0000479	PHI:419|CSH1|AAP93915|5476|Candida_albicans|Reduced_virulence
EVM0007454	PHI:1921|GzZC236|I1RCH8|5518|Gibberella_zeae_(related:_Fusarium_graminearum)|Reduced_virulence
EVM0002401	PHI:2100|Spf1|MGG_12005|148305|Magnaporthe_oryzae_(related:_Magnaporthe_grisea)|Mixed_outcome
EVM0001521	PHI:1511|GzHOME009|I1RXF3|5518|Gibberella_zeae_(related:_Fusarium_graminearum)|Reduced_virulence
EVM0004669	CHSF|PHI:3050|Q4WC58|746128|Aspergillus_fumigatus|mixed_outcome_

EVM0004891	PHI:432|FGL1|AAQ23181|5518|Fusarium_graminearum|Reduced_virulence
EVM0003159	PHI:2533|FKS1|Q4WLT4|746128|Aspergillus_fumigatus|Lethal
EVM0008462	PHI:1405|GzC2H069|I1RWW2|5518|Gibberella_zeae_(related:_Fusarium_graminearum)|Unaffected_pathogenicity
EVM0001555	PHI:881|MGG_04556|EDJ96020|318829|Magnaporthe_oryzae|Reduced_virulence
EVM0003299	PHI:578|NoxR|BAF36501|35717|Epichloe_festucae|Enhanced_antagonism
EVM0004556	PHI:2266|Ptr2|0|13684|Phaeosphaeria_nodorum_(related:_Stagonospora_nodorum)|Unaffected_pathogenicity
EVM0008074	PHI:2533|FKS1|Q4WLT4|746128|Aspergillus_fumigatus|Lethal
EVM0004814	PHI:2534|ERG11A|Q4WNT5|746128|Aspergillus_fumigatus|Mixed_outcome
EVM0008905	PHI:2406|MoSKN7|G4N7Y2|148305|Magnaporthe_oryzae_(related:_Magnaporthe_grisea)|Unaffected_pathogenicity
EVM0006845	PHI:2086|Moatg24|MGG_03638|148305|Magnaporthe_oryzae_(related:_Magnaporthe_grisea)|Unaffected_pathogenicity
EVM0003041	PHI:2158|MoCMK1|EU_984498|148305|Magnaporthe_oryzae_(related:_Magnaporthe_grisea)|Reduced_virulence
EVM0010231	PHI:511|CaNAG4|EAK93098|5476|Candida_albicans|Reduced_virulence
EVM0004644	PHI:2117|SPM1|P58371|148305|Magnaporthe_oryzae_(related:_Magnaporthe_grisea)|Reduced_virulence
EVM0003276	PHI:2042|ABC3|Q3Y5V5|148305|Magnaporthe_oryzae_(related:_Magnaporthe_grisea)|Loss_of_pathogenicity
EVM0004948	PHI:2293|cycA|B0XTA5|746128|Aspergillus_fumigatus|Reduced_virulence
EVM0008574	PHI:144|CHT42|AAC05829|29875|Trichoderma_virens|Reduced_virulence
EVM0005176	PHI:2293|cycA|B0XTA5|746128|Aspergillus_fumigatus|Reduced_virulence
EVM0002621	PHI:881|MGG_04556|EDJ96020|318829|Magnaporthe_oryzae|Reduced_virulence
EVM0006427	PHI:144|CHT42|AAC05829|29875|Trichoderma_virens|Reduced_virulence
EVM0004067	PHI:323|VFGLU1|AAO63562|93591|Verticillium_fungicola|Reduced_virulence
EVM0003563	PHI:144|CHT42|AAC05829|29875|Trichoderma_virens|Reduced_virulence
EVM0001189	PHI:267|MLT1|AAD51594|5476|Candida_albicans|Reduced_virulence
EVM0003856	PHI:1564|GzOB004|Q4IPZ1|5518|Gibberella_zeae_(related:_Fusarium_graminearum)|Unaffected_pathogenicity

EVM0004435	PHI:249|CYP1|AAG13968|318829|Magnaporthe_oryzae|Reduced_virulence
EVM0002609	PHI:2488|Man1|G4ND25|148305|Magnaporthe_oryzae_(related:_Magnaporthe_grisea)|Unaffected_pathogenicity
EVM0003992	PHI:249|CYP1|AAG13968|318829|Magnaporthe_oryzae|Reduced_virulence
EVM0008226	PHI:653|GIP2|AAL11721|67593|Phytophthora_sojae|Effector_(plant_avirulence_determinant)
EVM0006512	C14DM|PHI:3258|Q4QGX0|5664|Leishmania_major|reduced_virulence_
EVM0002377	PHI:144|CHT42|AAC05829|29875|Trichoderma_virens|Reduced_virulence
EVM0000213	PHI:1506|GzHOME001|I1RC01|5518|Gibberella_zeae_(related:_Fusarium_graminearum)|Unaffected_pathogenicity
EVM0002403	PHI:2081|Moatg15|MGG_12828|148305|Magnaporthe_oryzae_(related:_Magnaporthe_grisea)|Loss_of_pathogenicity
EVM0003064	PHI:267|MLT1|AAD51594|5476|Candida_albicans|Reduced_virulence
EVM0008611	PHI:397|LAC2|AAV64894|5207|Cryptococcus_neoformans|Unaffected_pathogenicity
EVM0000693	GGT|PHI:3146|O25743|210|Helicobacter_pylori|unaffected_pathogenicity_
EVM0008849	PHI:2117|SPM1|P58371|148305|Magnaporthe_oryzae_(related:_Magnaporthe_grisea)|Reduced_virulence
EVM0001903	PHI:432|FGL1|AAQ23181|5518|Fusarium_graminearum|Reduced_virulence
EVM0006554	PHI:2644|thioredoxin_1|P0AA28|90371|Salmonella_enterica_serovar_Typhimurium|Reduced_virulence
EVM0001722	PHI:432|FGL1|AAQ23181|5518|Fusarium_graminearum|Reduced_virulence
EVM0003767	PHI:2482|AlHK1|Q09JB7|160389|Alternaria_longipes|Increased_virulence_(Hypervirulence)
EVM0006123	PHI:2128|MoHox1|MGG_04853|148305|Magnaporthe_oryzae_(related:_Magnaporthe_grisea)|reduced_virulence
EVM0007566	PHI:2533|FKS1|Q4WLT4|746128|Aspergillus_fumigatus|Lethal
EVM0002356	PHI:1494|MAT1-1-3|I1RX41|5518|Gibberella_zeae_(related:_Fusarium_graminearum)|Unaffected_pathogenicity
EVM0003636	PHI:811|MGG_10510|EDK06580|318829|Magnaporthe_oryzae|Reduced_virulence
EVM0008542	PHI:2545|TIF35|Q4X1I3|746128|Aspergillus_fumigatus|Mixed_outcome
EVM0001357	PHI:440|PMR1|CAB87245|5476|Candida_albicans|Reduced_virulence
EVM0003013	PHI:2128|MoHox1|MGG_04853|148305|Magnaporthe_oryzae_(related:_Magnaporthe_grisea)|reduced_virulence

EVM0008015	PHI:1161|MgMfs1|A4ZGP3|54734|Mycosphaerella_graminicola_(related:_Zymoseptoria_tritici)|Chemistry_target
EVM0009816	PHI:2510|msdS/AfmsdC|Q6PWQ1|746128|Aspergillus_fumigatus|Unaffected_pathogenicity
EVM0003166	PHI:652|GIP1|AAL11720|67593|Phytophthora_sojae|Effector_(plant_avirulence_determinant)
EVM0009054	ssp|PHI:3418|Q5DPX0|29385|Staphylococcus_saprophyticus|mixed_outcome_
EVM0003445	PHI:1052|PHL1|ACB38886|135779|Cercospora_zeae-maydis|Unaffected_pathogenicity
EVM0002682	PHI:2400|Fgp2|I1S215|5518|Gibberella_zeae_(related:_Fusarium_graminearum)|Unaffected_pathogenicity
EVM0002472	PHI:2533|FKS1|Q4WLT4|746128|Aspergillus_fumigatus|Lethal
EVM0001087	PHI:2039|Con7p|Q069J4|148305|Magnaporthe_oryzae_(related:_Magnaporthe_grisea)|Loss_of_pathogenicity
EVM0005001	Vsp|PHI:3018|B7VSJ8|212663|Vibrio_splendidus|reduced_virulence_
EVM0000484	PHI:2533|FKS1|Q4WLT4|746128|Aspergillus_fumigatus|Lethal
EVM0007607	PHI:1816|GzZC131|I1RRS3|5518|Gibberella_zeae_(related:_Fusarium_graminearum)|Unaffected_pathogenicity
EVM0006745	PHI:2272|CpkB|A9Z1V6|13684|Phaeosphaeria_nodorum_(related:_Stagonospora_nodorum)|Unaffected_pathogenicity
EVM0004713	Pop1|PHI:3209|A4KTQ9|312341|Ceratocystis_populicola|reduced_virulence_
EVM0002111	PHI:816|MGG_04582|EDJ95999|318829|Magnaporthe_oryzae|Reduced_virulence
EVM0008189	PHI:811|MGG_10510|EDK06580|318829|Magnaporthe_oryzae|Reduced_virulence
EVM0004224	PHI:2533|FKS1|Q4WLT4|746128|Aspergillus_fumigatus|Lethal
EVM0007154	PHI:1301|GzbHLH002|I1RB45|5518|Gibberella_zeae_(related:_Fusarium_graminearum)|Unaffected_pathogenicity
EVM0004161	PHI:503|COX15|AAB64668|4932|Saccharomyces_cerevisiae|Reduced_virulence
EVM0002963	PHI:1301|GzbHLH002|I1RB45|5518|Gibberella_zeae_(related:_Fusarium_graminearum)|Unaffected_pathogenicity
EVM0001554	PHI:438|BcBOT1_(related:_CND5)|AAQ16576|40559|Botrytis_cinerea|Reduced_virulence
EVM0009437	PHI:1343|GzC2H003|I1RAF0|5518|Gibberella_zeae_(related:_Fusarium_graminearum)|Reduced_virulence
EVM0000556	PHI:881|MGG_04556|EDJ96020|318829|Magnaporthe_oryzae|Reduced_virulence
EVM0007007	PHI:871|MGG_12656|EDK01997|318829|Magnaporthe_oryzae|Reduced_virulence
EVM0001764	PHI:2533|FKS1|Q4WLT4|746128|Aspergillus_fumigatus|Lethal

EVM0006956	PHI:373|PLD1|EAK93902|5476|Candida_albicans|Reduced_virulence
EVM0004888	PHI:2349|VE1_(VEA)_(velvet)|G8CYZ6|5518|Gibberella_zeae_(related:_Fusarium_graminearum)|Reduced_virulence
EVM0005411	PHI:881|MGG_04556|EDJ96020|318829|Magnaporthe_oryzae|Reduced_virulence
EVM0000067	PHI:881|MGG_04556|EDJ96020|318829|Magnaporthe_oryzae|Reduced_virulence
EVM0004407	PHI:2519|LYS9|Q4WQ27|746128|Aspergillus_fumigatus|Lethal
EVM0005673	PHI:1071|Gas1|CAF05793|5270|Ustilago_maydis|Loss_of_pathogenicity
EVM0008273	PHI:1541|GzFlbD|I1RE42|5518|Gibberella_zeae_(related:_Fusarium_graminearum)|Unaffected_pathogenicity
EVM0009965	PHI:871|MGG_12656|EDK01997|318829|Magnaporthe_oryzae|Reduced_virulence
EVM0000925	PHI:96|FAS2|AAA34345|5476|Candida_albicans|Loss_of_pathogenicity
EVM0003286	PHI:2544|PAB1|Q4WK03|746128|Aspergillus_fumigatus|Mixed_outcome
EVM0004950	PHI:2038|Mir1|MGG_02370|148305|Magnaporthe_oryzae_(related:_Magnaporthe_grisea)|Unaffected_pathogenicity
EVM0009621	PHI:601|orf48|AAL20303|216597|Salmonella_enterica|Unaffected_pathogenicity
EVM0001674	PHI:96|FAS2|AAA34345|5476|Candida_albicans|Loss_of_pathogenicity
EVM0007332	PHI:2601|Oligopeptidase_B|Q94795|5693|Trypanosoma_cruzi|Reduced_virulence
EVM0001689	PHI:1506|GzHOME001|I1RC01|5518|Gibberella_zeae_(related:_Fusarium_graminearum)|Unaffected_pathogenicity
EVM0005693	PHI:274|USX1|AAM22494|5207|Cryptococcus_neoformans|Loss_of_pathogenicity
EVM0001653	PHI:2222|Sho1|XP_759303|5270|Ustilago_maydis|Mixed_outcome
EVM0007042	PHI:748|um00446|Not_available|5270|Ustilago_maydis|Unaffected_pathogenicity
EVM0002020	PHI:2644|thioredoxin_1|P0AA28|90371|Salmonella_enterica_serovar_Typhimurium|Reduced_virulence
EVM0001805	PHI:2117|SPM1|P58371|148305|Magnaporthe_oryzae_(related:_Magnaporthe_grisea)|Reduced_virulence
EVM0002806	PHI:1295|FgStuA|I1S0A8|5518|Gibberella_zeae_(related:_Fusarium_graminearum)|Reduced_virulence
EVM0006840	PHI:2490|MCA|E9B636|5665|Leishmania_mexicana|Mixed_outcome
EVM0010219	FgERG3A|PHI:3035|I1RFM2|5518|Fusarium_graminearum|reduced_virulence_
EVM0003076	PHI:2976|CgOPT1|C6ZRH8|29905|Colletotrichum_gloeosporioides_f._sp._aeschynomenes|Reduced_virulence
EVM0005568	PHI:2008|HST1|EDJ98541|148305|Magnaporthe_oryzae_(related:_Magnaporthe_grisea)|Unaffected_pathogenicity

EVM0003561	PHI:2109|CNB|MGG_06933|148305|Magnaporthe_oryzae_(related:_Magnaporthe_grisea)|Mixed_outcome
EVM0001631	PHI:871|MGG_12656|EDK01997|318829|Magnaporthe_oryzae|Reduced_virulence
EVM0007761	PHI:2510|msdS/AfmsdC|Q6PWQ1|746128|Aspergillus_fumigatus|Unaffected_pathogenicity
EVM0000122	Pop1|PHI:3209|A4KTQ9|312341|Ceratocystis_populicola|reduced_virulence_
EVM0003025	PHI:2321|SidI|Q4WR83|746128|Aspergillus_fumigatus|Reduced_virulence
EVM0008744	PHI:1180|(Sc_Sat4)|I1RS32|5518|Gibberella_zeae_(related:_Fusarium_graminearum)|Reduced_virulence
EVM0004653	PHI:2038|Mir1|MGG_02370|148305|Magnaporthe_oryzae_(related:_Magnaporthe_grisea)|Unaffected_pathogenicity
EVM0005770	PHI:419|CSH1|AAP93915|5476|Candida_albicans|Reduced_virulence
EVM0009772	PHI:652|GIP1|AAL11720|67593|Phytophthora_sojae|Effector_(plant_avirulence_determinant)
EVM0010134	PHI:2544|PAB1|Q4WK03|746128|Aspergillus_fumigatus|Mixed_outcome
EVM0001137	PHI:96|FAS2|AAA34345|5476|Candida_albicans|Loss_of_pathogenicity
EVM0000187	PHI:2042|ABC3|Q3Y5V5|148305|Magnaporthe_oryzae_(related:_Magnaporthe_grisea)|Loss_of_pathogenicity
EVM0004080	Pop1|PHI:3209|A4KTQ9|312341|Ceratocystis_populicola|reduced_virulence_
EVM0004025	PHI:871|MGG_12656|EDK01997|318829|Magnaporthe_oryzae|Reduced_virulence
EVM0008974	PHI:2552|NOP4|Q4WNM3|746128|Aspergillus_fumigatus|Mixed_outcome
EVM0000205	PHI:1742|GzZC057|I1S7H5|5518|Gibberella_zeae_(related:_Fusarium_graminearum)|Unaffected_pathogenicity
EVM0007726	PHI:748|um00446|Not_available|5270|Ustilago_maydis|Unaffected_pathogenicity
EVM0003315	PHI:2038|Mir1|MGG_02370|148305|Magnaporthe_oryzae_(related:_Magnaporthe_grisea)|Unaffected_pathogenicity
EVM0010224	PHI:2643|CFAS|A4HTK3|5671|Leishmania_infantum|Reduced_virulence
EVM0010061	PHI:1255|FGSG_07745|I1RQ67|5518|Gibberella_zeae_(related:_Fusarium_graminearum)|Unaffected_pathogenicity
EVM0006879	PHI:438|BcBOT1_(related:_CND5)|AAQ16576|40559|Botrytis_cinerea|Reduced_virulence
EVM0008470	PHI:2158|MoCMK1|EU_984498|148305|Magnaporthe_oryzae_(related:_Magnaporthe_grisea)|Reduced_virulence
EVM0000351	PHI:871|MGG_12656|EDK01997|318829|Magnaporthe_oryzae|Reduced_virulence

EVM0007277	PHI:174|GNA1|BAA36496|5476|Candida_albicans|Reduced_virulence
EVM0001968	PHI:2510|msdS/AfmsdC|Q6PWQ1|746128|Aspergillus_fumigatus|Unaffected_pathogenicity
EVM0000750	Atf1|PHI:3808|I1S0C0|5518|Fusarium_graminearum_(related:_Gibberella_zeae)|effector_(plant_avirulence_determinant)
EVM0000062	PHI:2930|ctf2|J9MFF7|59765|Fusarium_oxysporum_f._sp._Lycopersici|Reduced_virulence
EVM0008877	PHI:1255|FGSG_07745|I1RQ67|5518|Gibberella_zeae_(related:_Fusarium_graminearum)|Unaffected_pathogenicity
EVM0004058	PHI:2042|ABC3|Q3Y5V5|148305|Magnaporthe_oryzae_(related:_Magnaporthe_grisea)|Loss_of_pathogenicity
EVM0002444	PHI:2644|thioredoxin_1|P0AA28|90371|Salmonella_enterica_serovar_Typhimurium|Reduced_virulence
EVM0004514	PHI:33|PHR1|AAA68196|5476|Candida_albicans|Reduced_virulence

*DEGs were significantly upregulated in both S vs. pre-S and S vs. post-S.

Table S10 Differential expression of selected genes related to secondary metabolite biosynthesis, transport, and catabolism during conidiation.

Internal ID	FPKM*			Log2(FC) †		PHI
	Pre-C	C	Post-C	C vs. pre-C	C vs. post-C	
**ABC transporter**						
EVM0003125	0.00	3.00	0.00	6.51	7.05	PHI:2042
EVM0004058	5.71	11.80	5.54	1.08	1.36	PHI:2042
EVM0000187	9.02	20.94	8.40	1.26	1.59	PHI:2042
EVM0003276	0.48	3.21	0.46	2.74	3.05	PHI:2042
EVM0003064	35.03	189.63	16.33	2.47	3.80	PHI:267
EVM0001189	34.69	210.88	36.30	2.64	2.80	PHI:267
**Alcohol dehydrogenase GroES-like**						
EVM0001555	0.21	2.13	0.03	3.10	5.26	PHI:881
EVM0002621	0.30	2.22	0.10	2.68	4.23	PHI:881
EVM0000556	0.49	1.78	0.06	1.80	4.61	PHI:881
EVM0000067	0.31	1.06	0.02	1.72	4.86	PHI:881
EVM0005411	1.27	4.32	0.44	1.72	3.31	PHI:881
**Cytochrome P450**						
EVM0003715	0.08	3.17	0.42	4.84	3.10	PHI:2357
EVM0002185	0.04	73.62	4.56	6.53	4.22	PHI:4194
EVM0006879	10.31	22.53	9.59	1.16	1.49	PHI:438
Co_147	6.23	12.54	6.30	1.06	1.26	/
EVM0009502	0.02	3.72	0.33	6.30	3.43	PHI:438
EVM0001327	0.03	1.65	0.17	4.48	3.19	PHI:4194
EVM0004394	0.04	14.15	2.03	7.69	3.01	PHI:4194
EVM0009549	0.08	10.82	3.89	6.73	1.73	PHI:4194
EVM0001554	18.15	64.35	27.86	1.86	1.48	PHI:438
Co_423	0.03	1.76	0.14	4.97	3.49	/
EVM0005592	0.28	3.49	0.52	3.46	2.88	PHI:4194
EVM0002960	0.27	13.76	6.94	5.49	1.25	PHI:4194
EVM0003421	0.00	12.09	0.08	8.77	6.96	PHI:4194
EVM0004379	0.00	3.30	0.24	7.10	3.77	PHI:4194
EVM0004814	14.66	103.77	31.34	2.84	1.98	PHI:2534
Co_207	0.07	1.37	0.44	2.72	1.49	/
**Multicopper oxidase**						
EVM0004492	0.13	14.31	0.52	6.28	4.77	PHI:397
EVM0008611	0.34	1.85	0.68	2.40	1.68	PHI:397
EVM0005600	0.53	7.57	0.41	3.73	4.37	PHI:2920

* The transcript level is expressed as fragments per kilobase per million mapped reads (FPKM).

† FC is the fold change (FC) of DEGs based on a FC of ≥ 2 and an FDR of < 0.01.

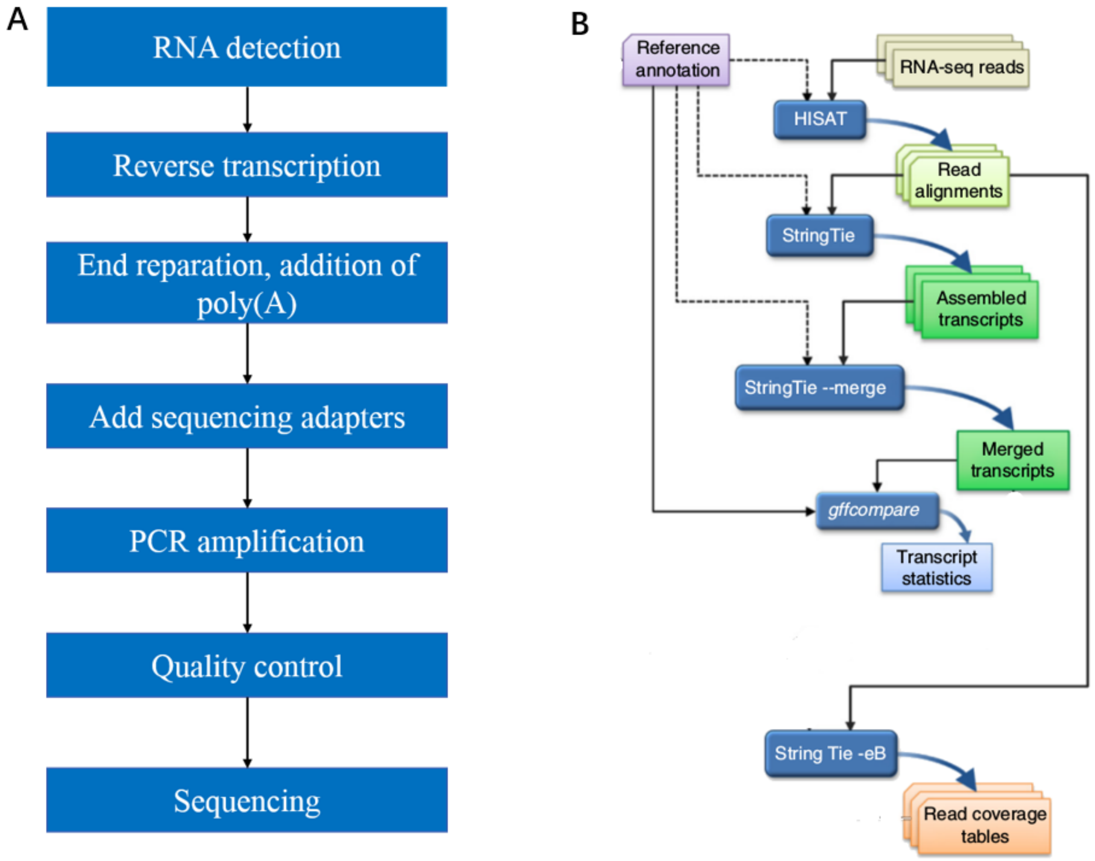

Figure S1. mRNA sequencing experimental pipeline (A) and the workflow of transcript assembly and statistics (B).

Figure S2. Characteristics of the de novo genome assembly. From the outside to the inside are as follows: I scaffolds, the different colors represent different scaffolds (the top10 scaffolds were chosen); II GC content; III gene density; IV miRNA; V tRNA.

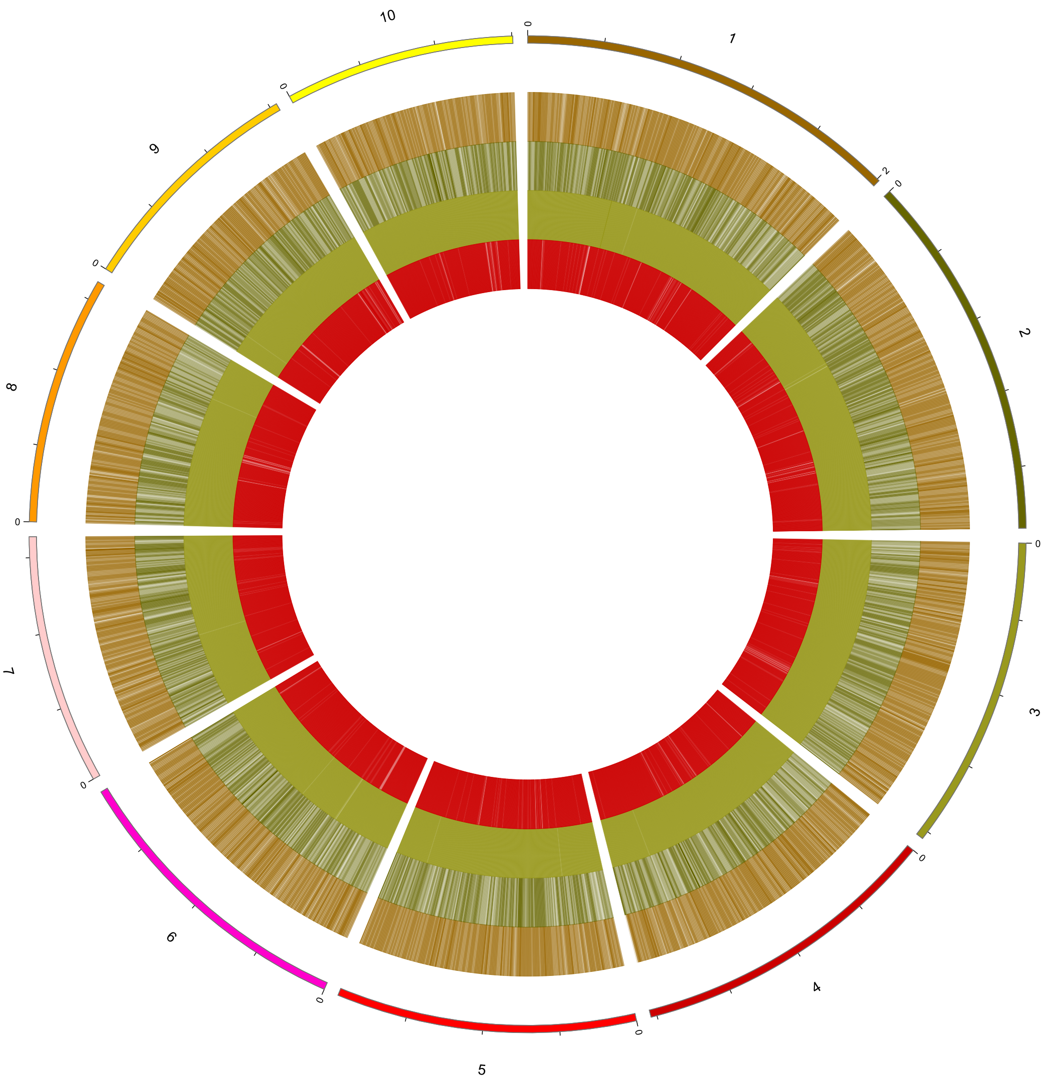

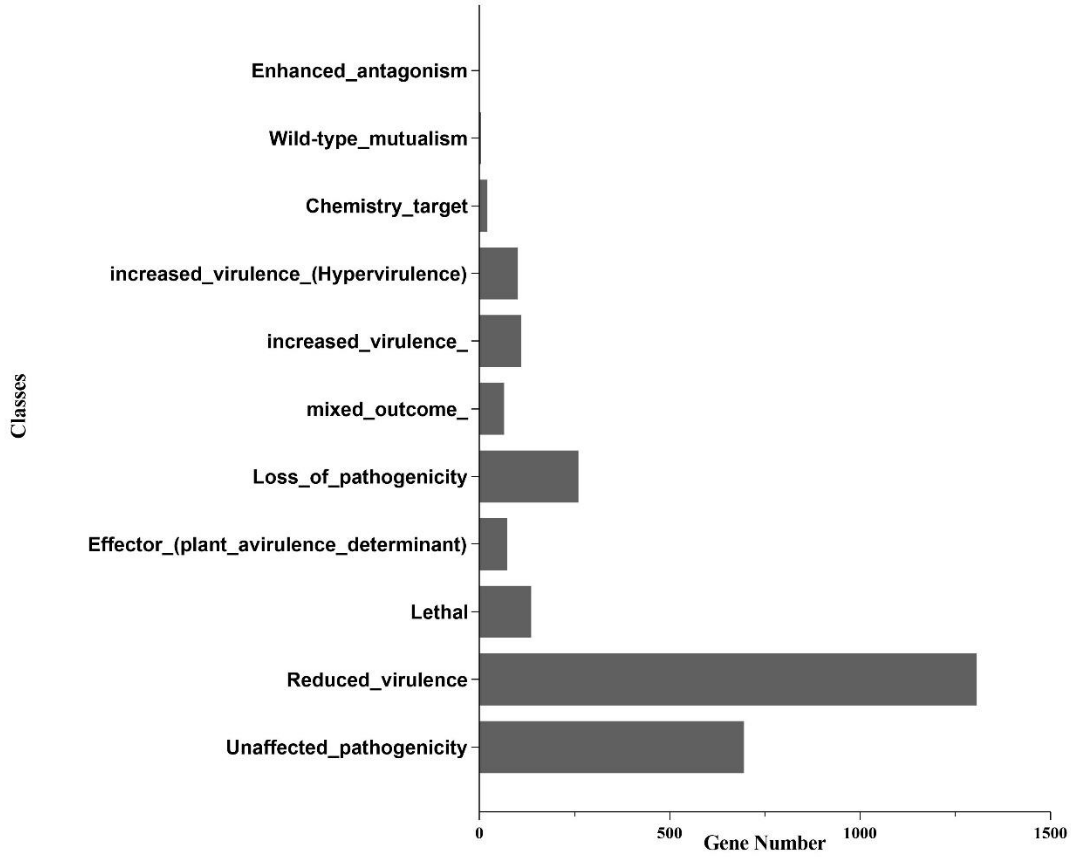

Figure S3. the PHI-annotated categories of the predicted protein-encoding genes of the *Conidiobolus obscurus* genome.

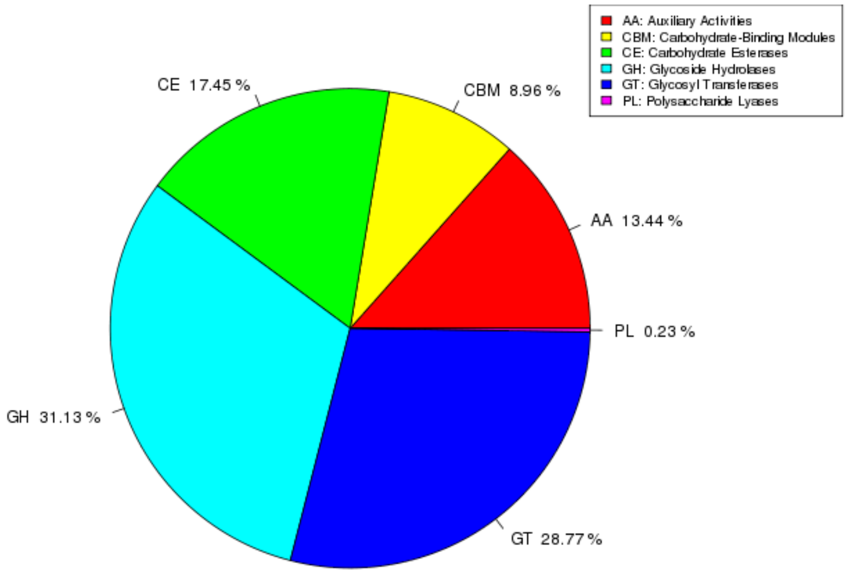

Figure S4. the CAZyme-coding genes in *Conidiobolus obscurus*.

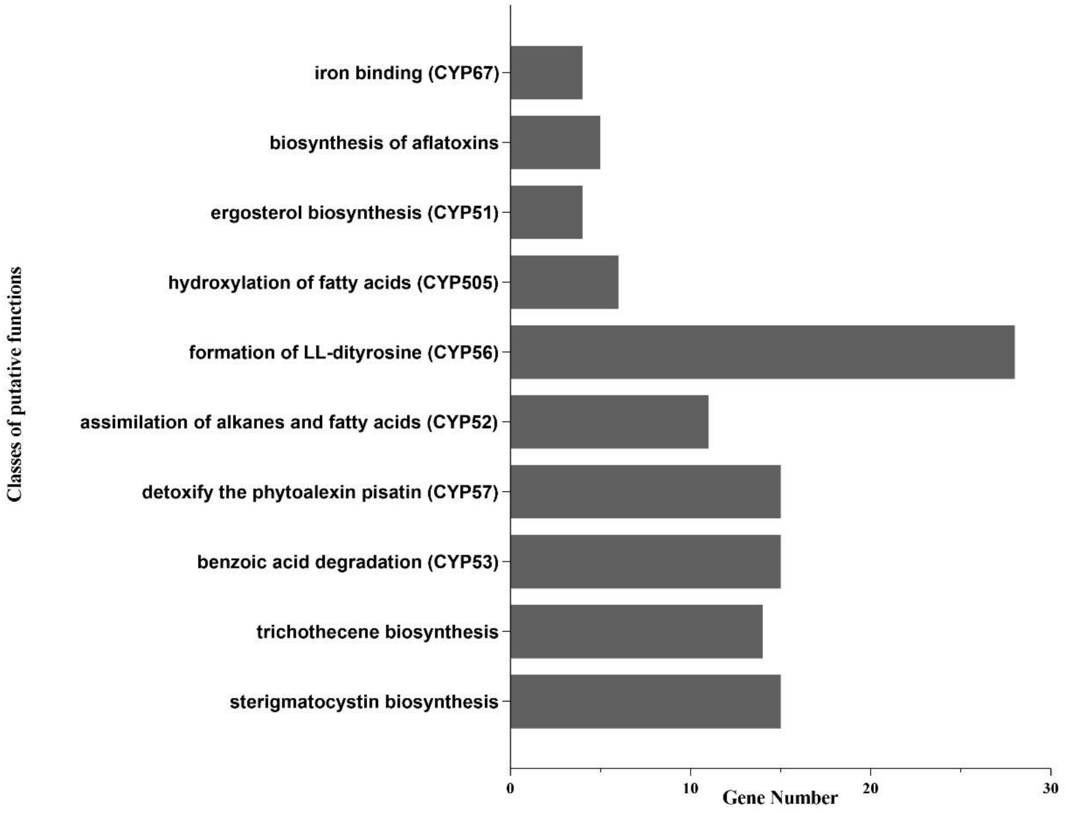

Figure S5. The putative functions and gene number of main P450 classes in the *Conidiobolus obscurus* genome.

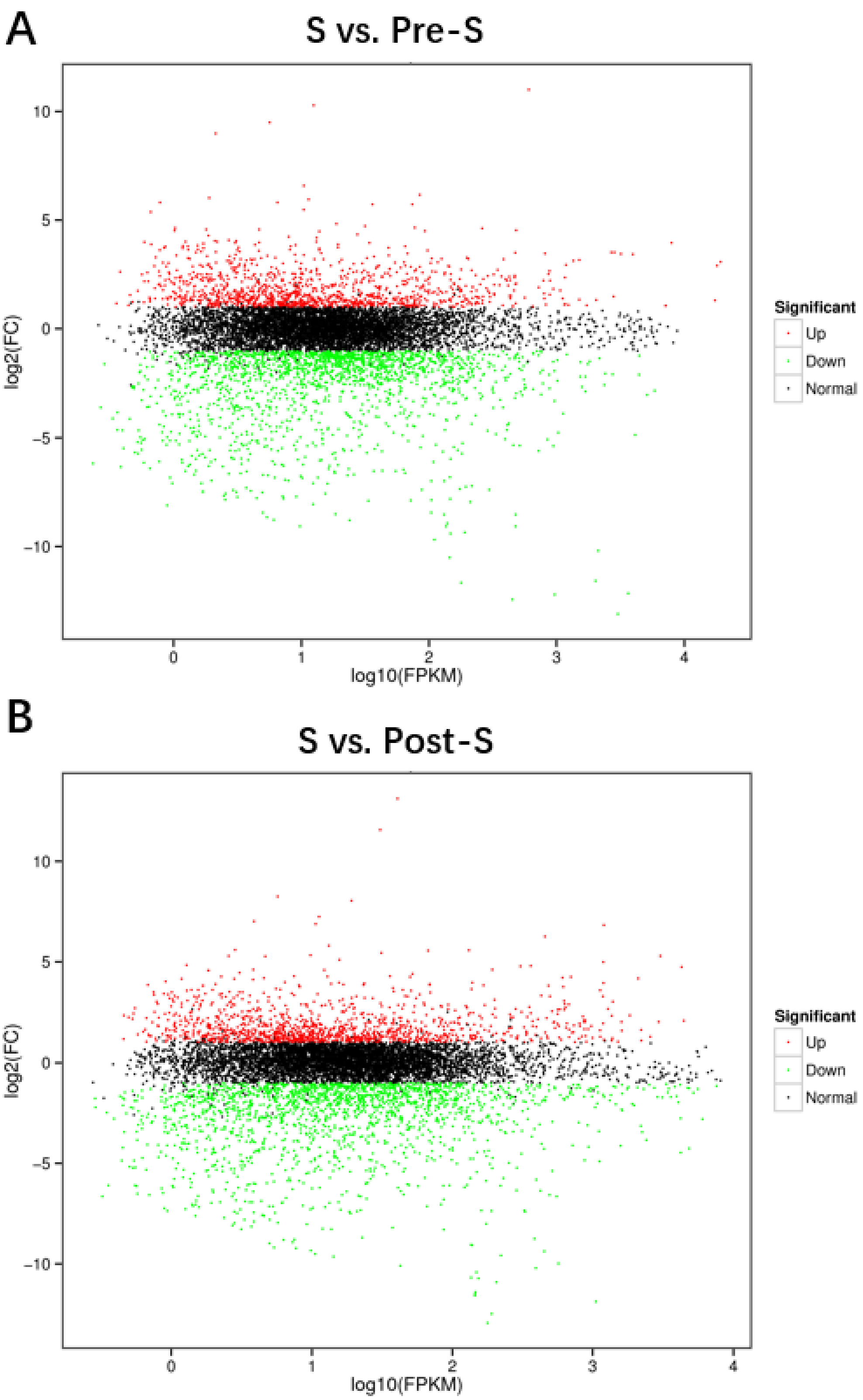

Figure S6. the MA plots of DEGs in C vs. Pre-C and C vs. Post-C.

Figure S7. Classification of upregulated genes in C stage vs. pre-C stage or C stage vs. post-C stage. (A) Number of upregulated genes putatively related to conidiation; (B) NCBI eukaryotic orthologous groups of proteins (KOG) functional annotation; (C) Gene Ontology (GO) functional annotation; (D) KEGG enrichment analysis. A smaller q value indicated a more significant enrichment, considering a FDR ≤ 0.01 as the threshold.

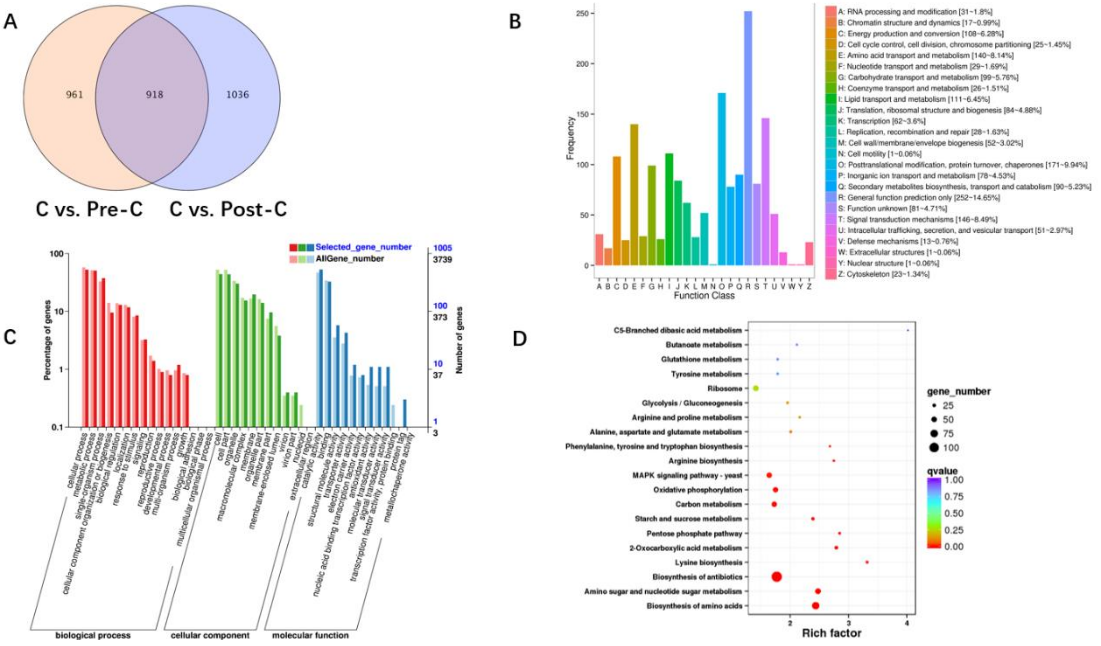

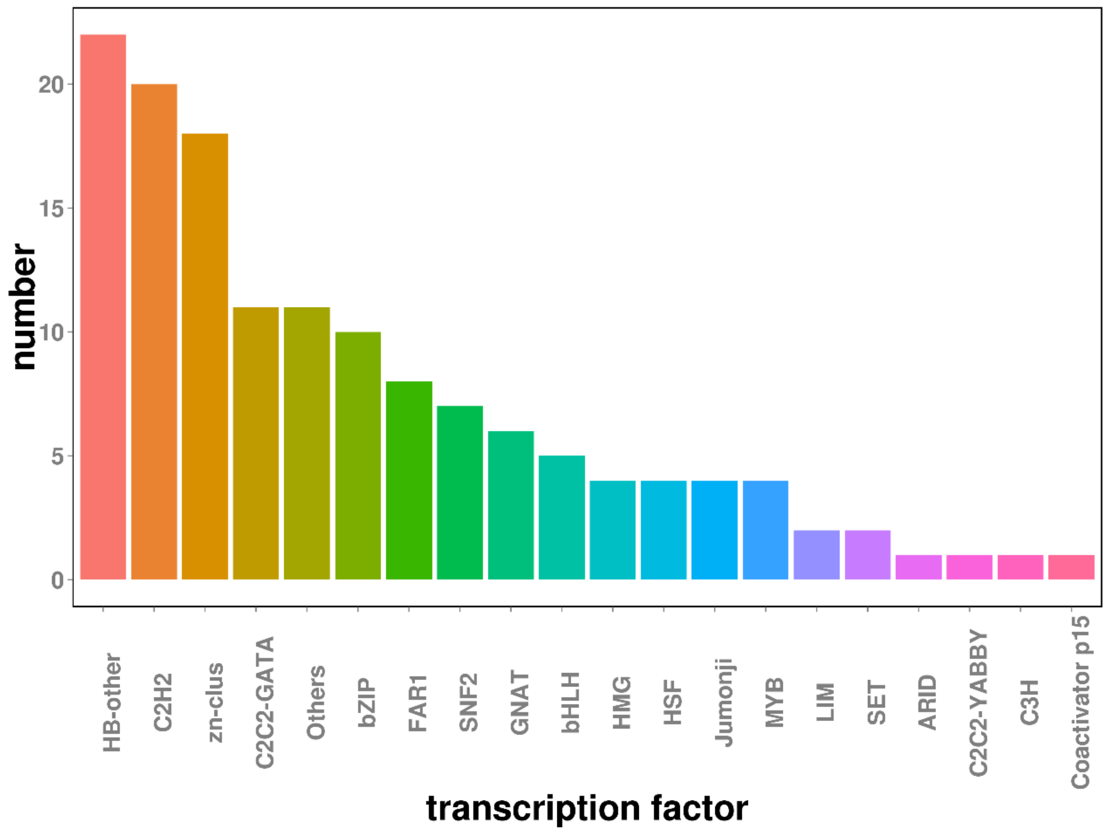

Figure S8. Transcription factor families

Figure S9. Validation of four differentially expressed transcripts at pre-C, C, and post-C stages of *C. obscurus* using RT-qPCR. (A) Heat shock transcription factor (EVM0008905); (B) Cytochrome P450 (EVM0004814); (C) β-glucan synthesis-associated protein (EVM0003971); (D) Trypsin-like serine proteinase (EVM0009373). Relative fold changes of C, post-C, were compared to the pre-C stage, which was set to a fold change of 1. Error bars are expressed as SEM from three biological replicates. Different lowercase letters marked on the bars indicated significant differences based on Fisher's least significant difference test following one-way ANOVA, with $P < 0.05$. The primers used are listed in Table S1.

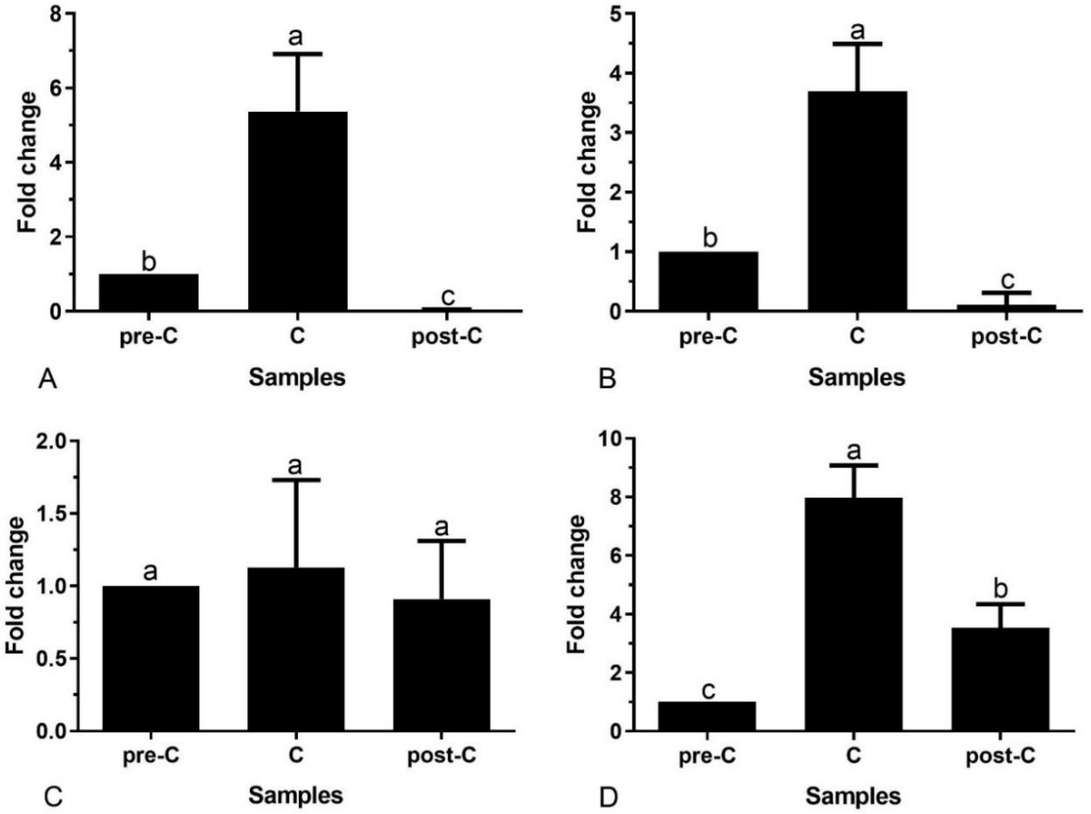

Supplement: Supplementary file 1 [file jof-08-00389-s001.zip › jof-1677922-supplementary.pdf]
